# ﻿*Rorippadaguanensis* (Brassicaceae), a new species from eastern China

**DOI:** 10.3897/phytokeys.253.145997

**Published:** 2025-03-11

**Authors:** Wei Zhang, Xiao-Yan Xiang, Ting-Shen Han, Jian-Wen Shao, Kai Zhao

**Affiliations:** 1 College of Life Sciences, Anqing Normal University, Anqing, Anhui 246011, China Anqing Normal University Anqing China; 2 CAS Key Laboratory of Tropical Forest Ecology, Xishuangbanna Tropical Botanical Garden, Chinese Academy of Sciences, Mengla, Yunnan 666303, China Xishuangbanna Tropical Botanical Garden, Chinese Academy of Sciences Mengla China; 3 College of Life Sciences, Anhui Normal University, Wuhu, Anhui 241000, China Anhui Normal University Wuhu China

**Keywords:** Morphology, new species, phylogenomics, *Rorippa*, taxonomy

## Abstract

*Rorippadaguanensis* W.Zhang & K.Zhao (Brassicaceae), a new species from Anhui and Hubei Provinces of eastern China, is described. Its floral morphology resembles *R.dubia*, with its fruit morphology similar to *R.cantoniensis*. However, it can be readily distinguished from *R.dubia* by its inconspicuous stems, oval silicle and from *R.cantoniensis* by the absence of petals and bracts on its flowers. The complete plastid genome of this new species is 155,594 bp in length. Phylogenetic analyses, based on whole plastid genome sequences of *Rorippa* species, revealed that *R.daguanensis* is sister to *R.cantoniensis*.

## ﻿Introduction

Brassicaceae is one of the largest angiosperm families, encompassing approximately 340 genera and more than 4140 species (İlçim 2008; Al-Shehbaz 2015; German et al. 2023). The genus *Rorippa* Scop. belongs to the Brassicaceae family, comprising approximately 86 species (Zheng et al. 2021; Han et al. 2024). These species are primarily distributed in the Northern Hemisphere and are found on every continent, except Antarctica (Stuckey 1972; Han et al. 2024; Ren et al. 2024).

According to the Flora of China, nine *Rorippa* species were recognised in China (Zhou et al. 2001). However, recent studies have documented new species and expanded distribution records for Chinese *Rorippa* (Zhang et al. 2009; Zheng et al. 2021). Currently, 11 *Rorippa* species are known to occur in China. The majority of these species are primarily found in subtropical lowland areas. Notably, *Rorippaelata* and *Rorippahengduanshanensis* are exceptions, being endemic to high-altitude regions of the Hengduan Mountains (HDM) (Zhou et al. 2001; Zheng et al. 2021).

**Figure 1.**
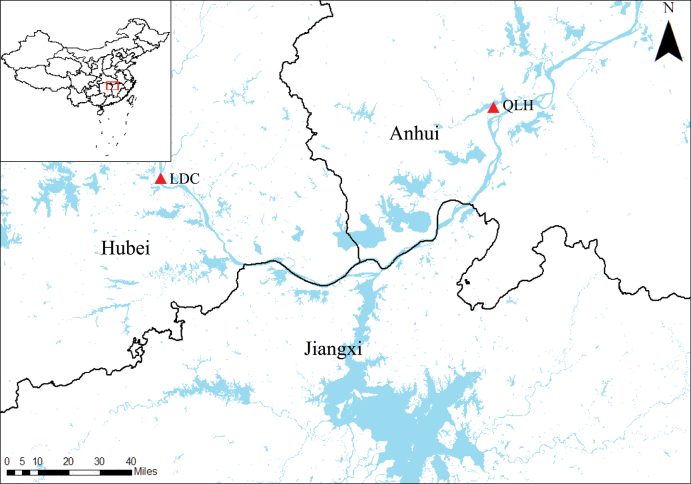
Known geographical distribution of *R.daguanensis* in China (red triangle).

During a field investigation in Qili Lake (QLH), Daguan District, Anqing City, Anhui Province, China, in April 2022 (Fig. 1), a novel *Rorippa* species was discovered. This unique plant exhibits inconspicuous stems, rosulate leaves, apetalous flowers lacking bracts and oval silicle. A comprehensive review of existing literature revealed no prior records of a *Rorippa* species possessing this combination of characteristics (Stuckey 1972; Al-Shehbaz 1991, 2015; Zhou et al. 2001; İlçim 2008). Careful morphological examination, coupled with molecular evidence, confirmed the discovery of a new species. This paper reports the results of this investigation and formally describes the new species as *Rorippadaguanensis* W.Zhang & K.Zhao.

## ﻿Materials and methods

### ﻿Sampling, morphological feature observation and comparison

Type specimens of the potential new species were collected in Qili Lake (QLH), (30°29'33.22"N, 116°54'31.27"E, alt. 7 m), Daguan District, Anqing City, Anhui Province (Fig. 1). They are deposited in the Herbarium of Herbarium of Anhui Normal University (ANUB). Morphological comparisons were performed between the new species and its closest relatives, *R.dubia* and *R.cantoniensis*. We examined both fresh materials and images from online resources, including the Chinese Virtual Herbarium (CVH, https://www.cvh.ac.cn/), the Global Biodiversity Information Facility (GBIF, https://www.gbif.org/) and the Plant Photo Bank of China (PPBC, http://ppbc.iplant.cn/). A total of seven diagnostic characteristics were used for the comparisons (Table 1).

**Table 1.** Morphological features comparison between *R.daguanensis* sp. nov. and its morphologically similar species.

Characters	*R.daguanensis*	*R.dubia*	*R.cantoniensis*
Plant height	3–8 cm	10–30 cm	10–30 cm
Stems	Inconspicuous	Conspicuous	Conspicuous
Basal leaves	Basal leaves do not wither during the flowering period	Basal leaves wither during the flowering period	Basal leaves wither during the flowering period
Petals	Absent	Absent	Obovate to narrowly spatulate, 2–3 (–3.5) × 0.5–1 mm
Bracts	Absent	Absent	Present throughout the raceme
Fruits	Silicle oval 5.5–6.5 mm × 2.5–3.5 mm	Silique linear 2.5–4 cm × 0.7–0.9 mm	Silicle oblong 4.5–8.5 mm × 1.5–2.5 mm
Pericarps	Wrinkled	Smooth	Smooth

### ﻿DNA extraction, chloroplast genome sequencing, assembly and annotation

Genomic DNA was extracted from silica gel-dried leaves using a modified CTAB protocol (Doyle and Doyle 1987). The DNA library construction and 150 bp paired-end sequencing were performed on the Illumina NovaSeq 6000 platform (Novogene, Tianjin, China), generating approximately 5 GB of raw data. The chloroplast genome of *R.daguanensis* was assembled using GetOrganelle v.1.7.5 (Jin et al. 2020), with *R.cantoniensis* chloroplast genome (ON892592) as a reference. The assembled chloroplast genome of *R.daguanensis* was annotated with Plastid Genome Annotator (PGA) (Qu et al. 2019). The CPGVIEW (www.1kmpg.cn/cpgview/) (Liu et al. 2023) was used to draw the circular map of the chloroplast genome. The sequences generated in this study were submitted to the NCBI database under accession numbers PQ159268 and OR992090.

### ﻿Phylogenetic analyses

To determine the phylogenetic position of the new species within the *Rorippa* genus, we downloaded 19 accessions of cp genome sequences of *Rorippa* species from NCBI for phylogenetic analysis. All sequences were aligned with MAFFT v.7 (Katoh and Standley 2013) with the default settings. A Maximum Likelihood (ML) phylogenetic tree was constructed using MEGA version 11.0.13 (Tamura et al. 2021) with 1000 bootstrap replicates. *Nasturtiumofficinale* (MK045962) and *Cardaminehirsuta* (MK637681) were used as outgroup taxa. The GTR+G+I model was selected as the best-fit substitution model.

## ﻿Results

### ﻿Characteristics of the complete plastid genome

**Figure 2.**
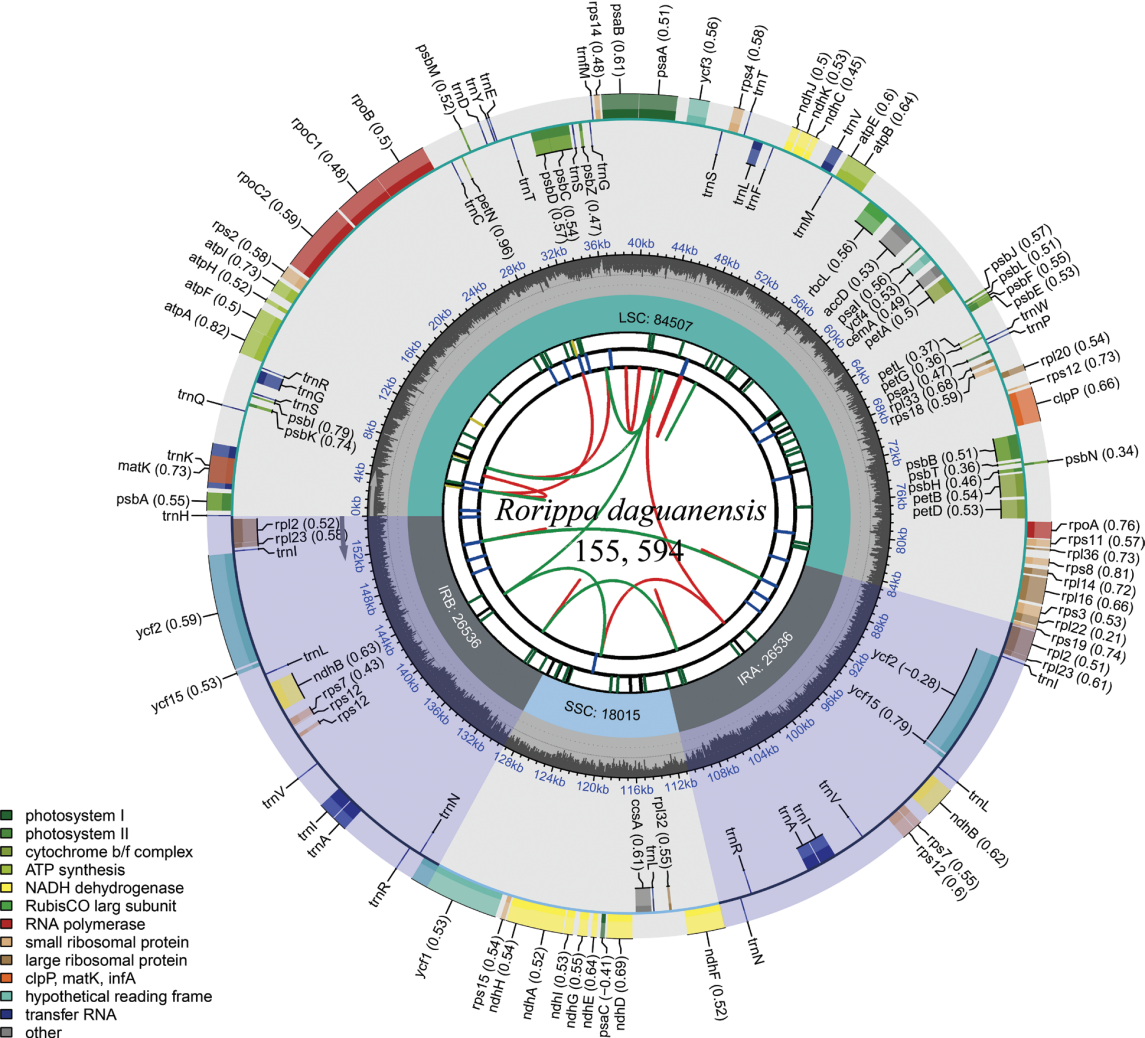
The complete chloroplast genome map of *R.daguanensis* sp. nov. The boxes of different sizes and colours on the outermost circle represent genes and their lengths. The inner and outer boxes of the outermost circle represent genes transcribed clockwise and counter-clockwise. The grey area in the middle circle represents the changes in GC content at different positions and the regions and lengths represented by the tetrad structure (LSC, SSC, IRa and IRb) are drawn in different colours on the inner circle.

The chloroplast genome of *R.daguanensis* was 155,594 bp in length and exhibited a typical quadripartite structure (Fig. 2). It comprised a large single-copy region (LSC of 84,507 bp), a small single-copy region (SSC of 18,015 bp) and a pair of inverted repeat regions (IRs of 26,536 bp in each) (Fig. 2). The GC content of the whole chloroplast genome was 36.3%. The *R.daguanensis* chloroplast genome encoded 130 genes, including 85 protein-coding genes (PCGs), 37 transfer RNA genes (tRNAs) and eight ribosomal RNA genes (rRNAs).

### ﻿Molecular phylogenetic relationship

**Figure 3.**
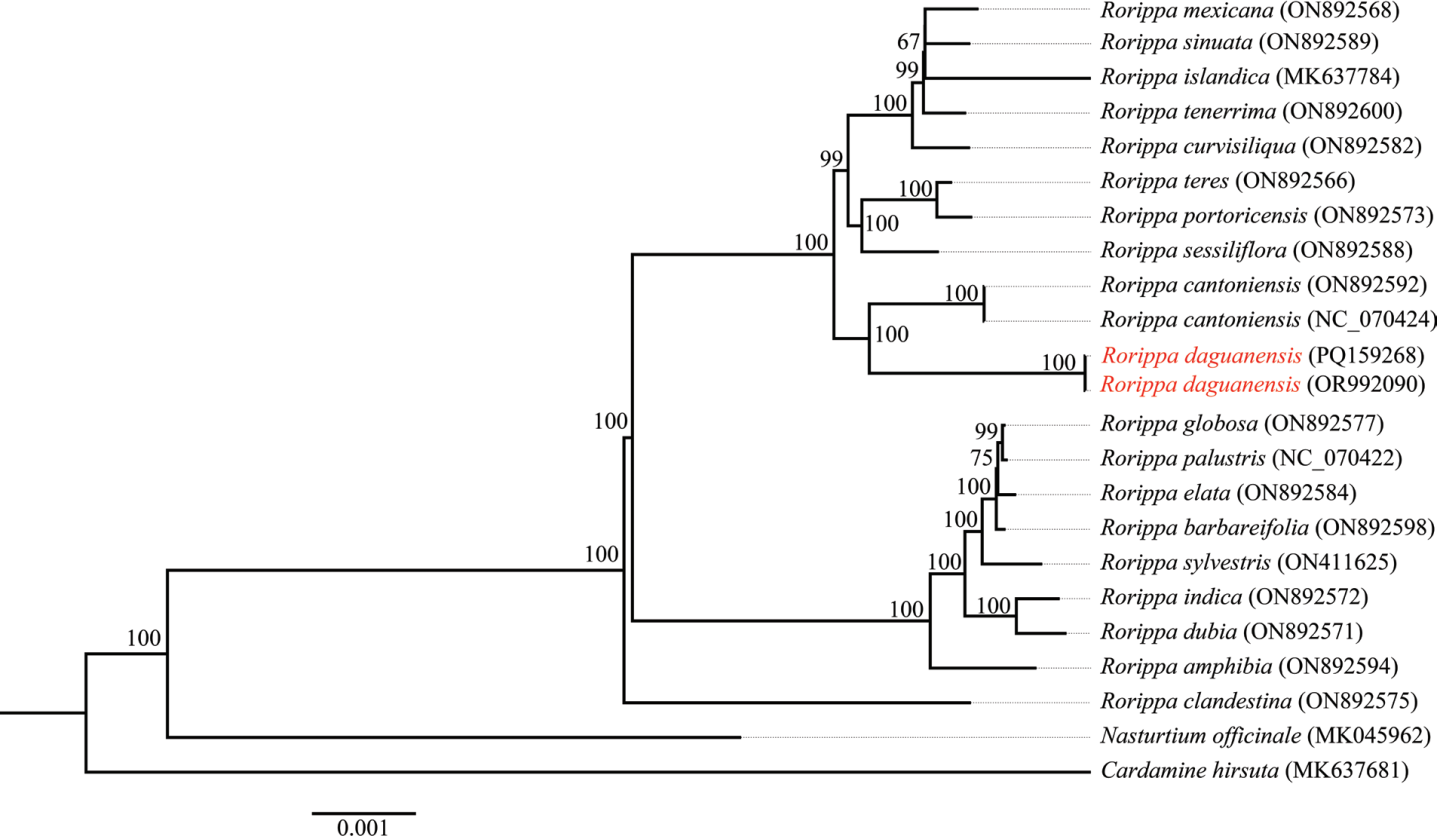
Phylogenetic placement of *R.daguanensis* using the Maximum Likelihood (ML) method, based on the whole plastid genome; bootstrap supports (BS) are shown on branches. NCBI accession numbers are shown in parentheses.

Phylogenetic analysis of the whole plastid genome using Maximum Likelihood (ML) revealed the phylogenetic relationship of the new species and its congeners. The results placed the new species within the genus *Rorippa* and the overall topology of *Rorippa* was congruent with previous studies (Nakayama et al. 2014, 2018; Zheng et al. 2021; Han et al. 2024). *R.daguanensis* was identified as the sister species of *R.cantoniensis*, with both species forming a well-supported monophyletic group (bootstrap support (BS) = 100%) (Fig. 3).

### ﻿Morphological comparison

In morphology, this new species resembles *R.dubia* in ﬂoral characters and is similar to *R.cantoniensis* in fruit characters. However, it can be readily distinguished from *R.dubia* by its inconspicuous stems, oval silicle and from *R.cantoniensis* by the absence of petals and bracts on its flowers. Detailed morphological comparisons between the new species and its morphologically similar species are summarised in Table 1.

### ﻿Taxonomic treatment

#### ﻿ Rorippa daguanensis

Taxon classificationPlantaeBrassicalesBrassicaceae

W.Zhang & K.Zhao sp. nov.

3CD10A05-C356-5C09-A289-49CE0B206A25

urn:lsid:ipni.org:names:77358194-1

Figs 4 , 5

##### Type.

China • Anhui, Anqing City, Daguan District, Qili Lake, 30°29'33.22"N, 116°54'31.27"E, alt. 7 m, 18 April 2022, *Wei Zhang & Kai Zhao* ZW20220418 (holotype: ANUB!; isotypes: ANUB!; isotypes: CSH!) (Fig. 5).

##### Diagnosis.

*Rorippadaguanensis* is similar to *R.dubia* and *R.cantoniensis*, but it can be readily distinguished from *R.dubia* by inconspicuous stems (vs. conspicuous stems), oval silicle 5.5–6.5 mm × 2.5–3.5 mm (vs. linear silique 2.5–4 cm × 0.7–0.9 mm) and can be differentiated from *R.cantoniensis* by the absence of petals and bracts on the flowers (vs. flowers with petals and bracts) and wrinkled pericarps (vs. smooth pericarps).

**Figure 4.**
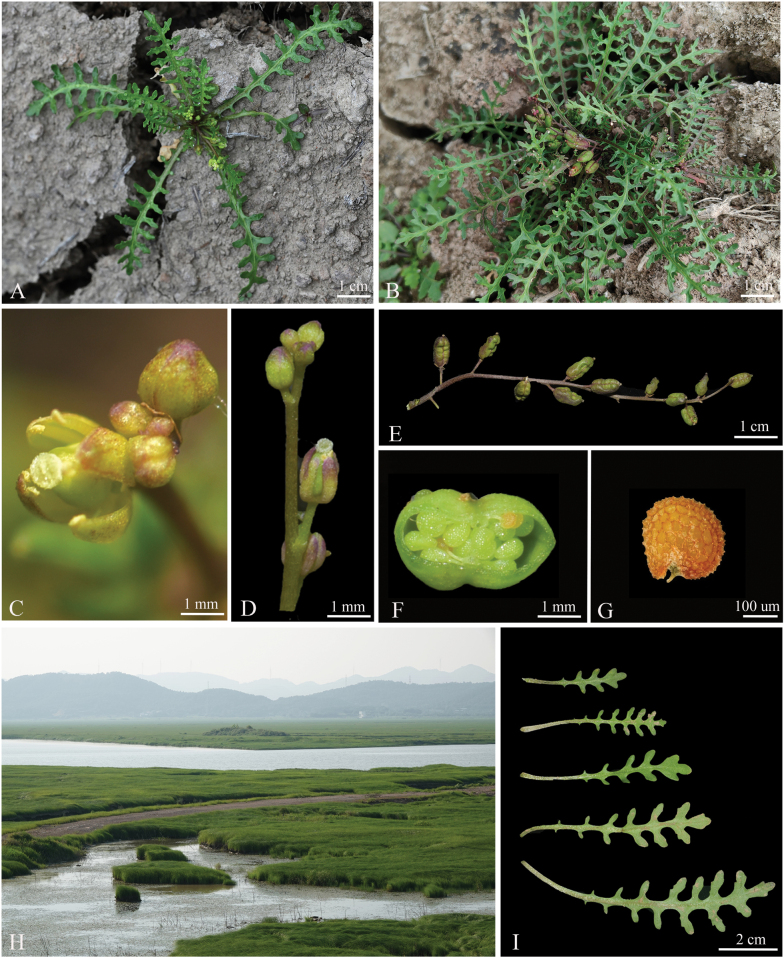
Morphological characters of *R.daguanensis* sp. nov. **A** plant in ﬂowering **B** plant in fruiting **C** apetalous flower **D** inflorescence **E** infructescence **F** transection of fruits **G** seed **H** habitat **I** leaf morphology.

##### Description.

Herbs annual, 3–8 cm tall, glabrous throughout. Stems mostly inconspicuous, with a few erect or decumbent. Leaves petiolate, 10–20, forming an open rosette, pinnatisect or almost bipinnatisect, 2–10 × 1–2 cm, lateral lobes 3–10 on each side of mid-vein, 2–10 × 1–4 mm, incised, dentate or entire. Racemes ebracteate. Fruiting pedicels stout, ascending, 1–4 mm. Sepals purple or green, ascending, oblong or subelliptic, 0.8–1.2 × 0.2–0.4 mm. Petals absent. Filaments 0.8–1.2 mm; anthers oblong, 0.1–0.3 mm. Ovules 100–200 per ovary. Fruit broadly or narrowly oblong, 5.5–6.5 × 2.5–3.5 mm; pericarp wrinkled, valves thin papery, veinless; style 1–1.4 mm. Seeds reddish-brown, ovate or oblate, foveolate, 0.2–0.4 × 0.2–0.4 mm.

**Figure 5.**
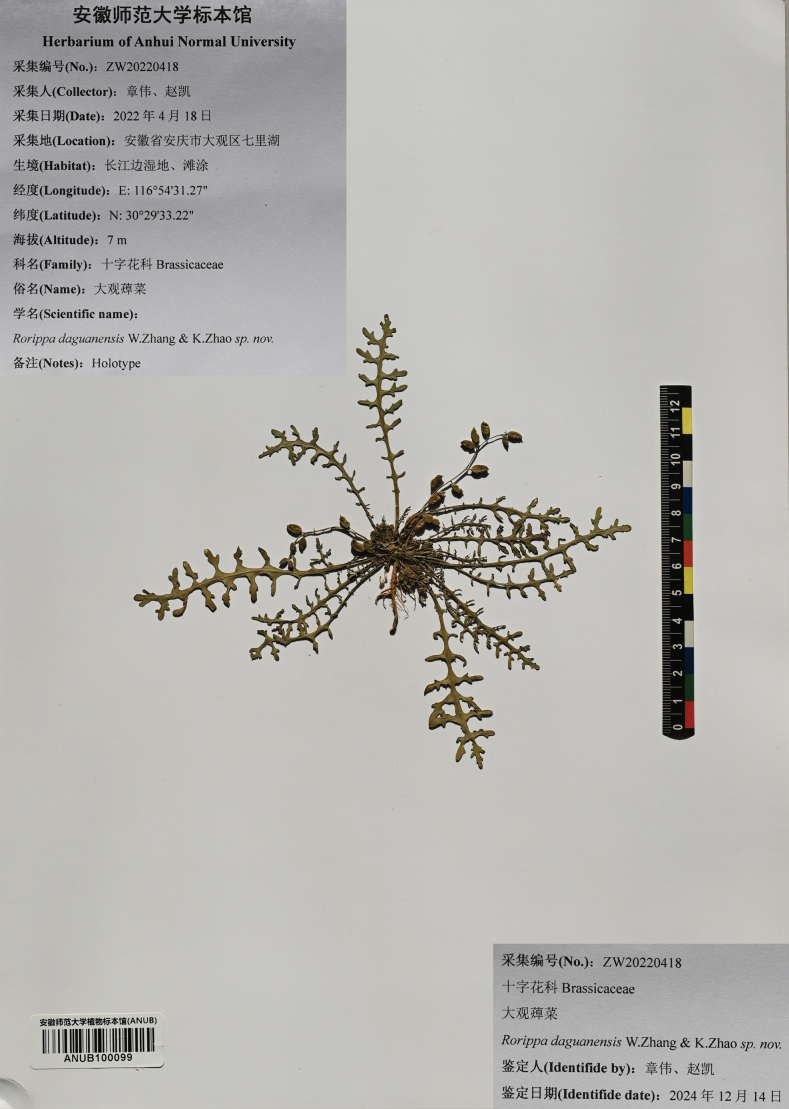
Holotype of *R.daguanensis* sp. nov. (ANUB100099).

##### Phenology.

Flowering and fruiting from February to November.

##### Chinese name.

Dà Guān Hān Cài (大观蔊菜).

##### Etymology.

The specific epithet ‘*daguanensis*’ refers to the type specimen collection locality, Daguan District, Anqing City, Anhui, China.

##### Distribution and ecology.

Based on our field survey, *Rorippadaguanensis* is currently known only from two populations: one located in Qili Lake (QLH), Daguan District, Anqing City, Anhui Province and the other in Lidu Village (LDC), Xishui County, Huanggang City, Hubei Province (Fig. 1). It grows in wetlands and mudflats along the Yangtze River, at elevations between 7 m and 13 m. This characteristic results in making *R.daguanensis* a well-defined obligate wetland indicator in its native area.

##### Preliminary conservation assessment.

Currently, only two populations of *Rorippadaguanensis* were recognised in eastern China, each consisting of approximately 300 individuals. The estimated Extent of Occurrence (EOO) for this species is 15 km^2^ and the Area of Occupancy (AOO) is 0.05 km^2^. In addition, its living environment has been significantly impacted by seasonal floods and human activities over an extended period. Therefore, based on IUCN Red List Categories and Criteria (IUCN 2024), the conservation status of this new species is evaluated as ‘Vulnerable’ (VU) as it meets criteria D1 and D2.

### ﻿Key to the species of *Rorippa* in China

**Table:** 

1	Racemes bracteate throughout or rarely along lowermost third	**2**
–	Racemes ebracteate, rarely lowermost 1 or 2 flowers bracteate	**3**
2	Fruiting pedicels slender, 3–6.5(-8) mm; fruit linear, 7–17(-21) × 1.2–1.6 mm; seeds colliculate	***R.benghalensis***
–	Fruiting pedicels stout, (0.3-)0.7–2(-3) mm; fruit oblong, (3-)4.5–8.5(-10) × 1.5–2.5 mm; seeds foveolate	***R.cantoniensis***
3	Fruiting pedicels erect to erect-ascending, subappressed to rachis; fruit valves with a distinct mid-vein; seeds 1.1–1.5 × 0.7–1.1 mm	***R.elata***
–	Fruiting pedicels ascending, divaricate or reflexed, not appressed to rachis; fruit valves not veined; seeds 0.4–0.9 × 0.3–0.6 mm	**4**
4	Fruit globose, oblong, ellipsoid or oblong-ovoid, length less than 3× width	**5**
–	Fruit linear, rarely linear-oblong, length more than 4 × width	**9**
5	Fruit oblong, ellipsoid or oblong-ovoid	**6**
–	Fruit globose or subglobose	**8**
6	Petals absent, Fruiting pedicels shorter than fruit	***R.daguanensis***
–	Petals present, Fruiting pedicels longer than fruit	**7**
7	Leaves dentate, Fruit not curved	***R.amphibia***
–	Leaves pinnatisect, Fruit often slightly curved	***R.palustris***
8	Fruit valves 2, papery; sepals (1-)1.3–1.8(-2) mm; petals 0.7–1.3(-1.5) × 0.3–0.8 mm	***R.globosa***
–	Fruit valves (3 or)4(-6), leathery; sepals 1.6–2.8 mm; petals (1.5-)1.8–3(-3.5) × 0.7–1.8(-2) mm	***R.barbareifolia***
9	Perennials; middle cauline leaves deeply pinnatisect; fruit rarely producing seeds; seeds colliculate	***R.sylvestris***
–	Annuals; middle cauline leaves lyrate-pinnatipartitite or undivided; fruit producing numerous seeds; seeds foveolate	**10**
10	Seeds biseriate; fruit often 1–2.5 cm long, 1–1.5 mm wide	***R.indica***
–	Seeds uniseriate; fruit often 2–3.5 cm long, 0.5–1 mm wide	**11**
11	Petals mostly absent, if present, often shorter than sepals, 1.5–2.5 mm long, 0.2–0.7(–1) mm wide; fruit straight, (1.5–) 2.5–3.5(–4) cm long, 0.7–0.9 (–1) mm wide	***R.dubia***
–	Petals present, petals longer than sepals or equal to the sepals, (1.5–)2–3(–3.5) mm long, 1–2 mm wide; fruit often curved, (1.5–)2–3(–3.5) cm long, 0.5–0.7 (–8) mm wide	***R.hengduanshanensis***

## Supplementary Material

XML Treatment for Rorippa daguanensis

## ﻿Additional information

### Author ORCIDs

Wei Zhang https://orcid.org/0000-0002-0488-7564

Xiao-Yan Xiang https://orcid.org/0009-0004-7945-5653

Ting-Shen Han https://orcid.org/0000-0002-8612-6581

Kai Zhao https://orcid.org/0009-0001-3601-0652

### Data availability

All of the data that support the findings of this study are available in the main text.

## References

[B1] Al-Shehbaz IA (1991) *Rorippabeckii* (Brassicaceae), a new species from Bolivia. Novon 1(1): 9–11. 10.2307/3391710

[B2] Al-Shehbaz IA (2015) Brassicaceae. In: Hong DY (Ed.) Flora of the Pan-Himalaya. Vol. 30. Science Press, Beijing & Cambridge University Press, Cambridge, 595 pp.

[B3] Doyle JJ Doyle JL (1987) A rapid DNA isolation procedure for small quantities of fresh leaf tissue. Phytochemical Bulletin 19(1): 11–15.

[B4] German DA Hendriks KP Koch MA Lens F Lysak MA Bailey CD Mummenhoff K Al-Shehbaz IA (2023) An updated classification of the Brassicaceae (Cruciferae). PhytoKeys 220: 127–144. 10.3897/phytokeys.220.97724 37251613 PMC10209616

[B5] Han TS Yu CC Zheng QJ Kimura S Onstein RE Xing YW (2024) Synergistic polyploidization and long-distance dispersal enables the global diversification of yellowcress herbs. Global Ecology and Biogeography 33(3): 458–469. 10.1111/geb.13798

[B6] İlçim A (2008) *Rorippabehcetii* (Brassicaceae), a New Species from Turkey. Annales Botanici Fennici 45(6): 485–487. 10.5735/085.045.0608

[B7] IUCN (2024) Guidelines for using the IUCN red list categories and criteria. Version 16. Prepared by the Standards and Petitions committee. http://www.iucnredlist.org/documents/RedListGuidelines.pdf

[B8] Jin JJ Yu WB Yang JB Song Y Li DZ (2020) GetOrganelle: A fast and versatile toolkit for accurate de novo assembly of organelle genomes. Genome Biology 21(1): 241. 10.1186/s13059-020-02154-5 32912315 PMC7488116

[B9] Katoh K Standley DM (2013) MAFFT multiple sequence alignment software version 7: Improvements in performance and usability. Molecular Biology and Evolution 30(4): 772–780. 10.1093/molbev/mst010 23329690 PMC3603318

[B10] Liu S Ni Y Li J Zhang X Yang H Chen H Liu C (2023) CPGView: A package for visualizing detailed chloroplast genome structures. Molecular Ecology Resources 23(3): 694–704. 10.1111/1755-0998.13729 36587992

[B11] Nakayama H Fukushima K Fukuda T Yokoyama J Kimura S (2014) Molecular Phylogeny Determined Using Chloroplast DNA Inferred a New Phylogenetic Relationship of *Rorippaaquatica* (Eaton) EJ Palmer & Steyermark (Brassicaceae)—Lake Cress. American Journal of Plant Sciences 5(1): 48–54. 10.4236/ajps.2014.51008

[B12] Nakayama H Sakamoto T Okegawa Y Kaminoyama K Fujie M Ichihashi Y Kurata T Motohashi K Al-Shehbaz I Sinha N Kimura S (2018) Comparative transcriptomics with self-organizing map reveals cryptic photosynthetic differences between two accessions of North American Lake cress. Scientific Reports 8(1): 3302. 10.1038/s41598-018-21646-w 29459626 PMC5818620

[B13] Qu XJ Moore MJ Li DZ Yi TS (2019) PGA: A software package for rapid, accurate, and flexible batch annotation of plastomes. Plant Methods 15(1): 50. 10.1186/s13007-019-0435-7 31139240 PMC6528300

[B14] Ren T Xun L Jia Y Li B (2024) Complete plastomes of ten *Rorippa* species (Brassicaceae): Comparative analysis and phylogenetic relationships. Agronomy 14(5): 913. 10.3390/agronomy14050913

[B15] Stuckey RL (1972) Taxonomy and distribution of the genus *Rorippa* (Cruciferae) in North America. SIDA, Contributions to Botany 4: 279–443. https://biostor.org/reference/158488

[B16] Tamura K Stecher G Kumar S (2021) MEGA11: Molecular evolutionary genetics analysis version 11. Molecular Biology and Evolution 38(7): 3022–3027. 10.1093/molbev/msab120 33892491 PMC8233496

[B17] Zhang SM Li ZX Wang Q Chen C Jiang XP Sun HK (2009) A Newly Recorded Species *Rorippaamphibia* (L.) Besser from China. Redai Yaredai Zhiwu Xuebao 17(2): 176–178. 10.3969/j.issn.1005-3395.2009.2.2112

[B18] Zheng QJ Yu CC Xing Y Han T-S (2021) A new *Rorippa* species (Brassicaceae), *R.hengduanshanensis*, from the Hengduan Mountains in China. Phytotaxa 480(3): 210–222. 10.11646/phytotaxa.480.3.1

[B19] Zhou TY Lu LL Yang G Al-Shehbaz IA (2001) Brassicaceae. In: Wu ZY, Raven PH, Hong DY (Eds) Flora of China. Science Press, Beijing & Missouri Botanical Garden Press, St. Louis, 506 pp.

